# Measuring attraction to screen devices in early childhood: development of the Affinity-TV and Affinity-Mobile scales

**DOI:** 10.3389/fped.2025.1496225

**Published:** 2025-03-05

**Authors:** Darcy A. Thompson, Laura K. Kaizer, Sarah J. Schmiege, Natasha J. Cabrera, Lauren Clark, Haley Ringwood, Estefania Miramontes Valdes, Jeanne M. Tschann

**Affiliations:** ^1^ Department of Pediatrics, School of Medicine, University of Colorado Anschutz Medical Campus, Aurora, CO, United States; ^2^ Adult & Child Center for Outcomes Research & Delivery Science, University of Colorado Anschutz Medical Campus, Aurora, CO, United States; ^3^ Department of Biostatistics and Informatics, Colorado School of Public Health, University of Colorado Anschutz Medical Campus, Aurora, CO, United States; ^4^ College of Education, University of Maryland, College Park, MD, United States; ^5^ School of Nursing, University of California, Los Angeles, CA, United States; ^6^ Department of Family Medicine, Denver Health and Hospital Authority, Denver, CO, United States; ^7^ Department of Family Medicine, School of Medicine, University of Colorado Anschutz Medical Campus, Aurora, CO, United States; ^8^ Department of Psychiatry and Behavioral Sciences, University of California, San Francisco, CA, United States

**Keywords:** digital media, infants, toddlers, behavioral response, screen device, problematic screen use, Latino (Hispanic)

## Abstract

**Introduction:**

With the increasing integration of digital screen devices into our everyday life, there has been increased attention regarding the risk of “problematic” use or pathological use. Because children start using screen devices in the first few years of life, early identification of those at risk for future problematic use could inform early prevention efforts. Children's attraction to screen devices in early childhood may identify those at risk for future problematic use; however currently, there are no measures of toddlers' attraction or affinity to screen devices. The objective of this study was to develop survey measures of toddler affinity to screen media, inclusive of televisions, smartphones, and tablets.

**Methods:**

Measures were developed using an exploratory sequential mixed methods (qualitative -> quantitative) approach. Participants were Mexican American mothers of toddlers 15–26 months old. Findings from semi-structured interviews were used to develop items reflecting parental reports of child affinity to screen devices. Items were administered by phone to 384 mothers. Analyses included evaluation of the factor structure and psychometric properties of Affinity-TV (10 items) and Affinity-Mobile (12 items), and evaluations of correlations between each scale with social emotional outcomes and demographic characteristics.

**Results:**

Factor analysis supported a one-factor solution for each scale. Reliabilities were acceptable for both scales (Cronbach's alpha > .75). There was a significant positive correlation between Affinity-TV and Affinity-Mobile (rs = 0.44, *p* < 0.001). Affinity-TV was significantly positively correlated with toddler average daily minutes of TV use (rs = 0.27, *p* < 0.001) and average daily minutes of mobile use (rs = 0.10, *p* < 0.05). Affinity-Mobile was significantly positively correlated with toddler average daily minutes of mobile use (rs = 0.31, *p* < 0.001), but not with average daily minutes of TV (rs = −0.04, NS). Each scale was correlated with social emotional developmental outcomes.

**Discussion:**

The Affinity-TV and Affinity-Mobile scales have good initial reliability and adequate predictive validity. These findings support the use of Affinity-TV and Affinity-Mobile in toddlers as measures of children's attraction to screen devices. These measures may help to identify early risk for problematic use, and they offer a novel way to evaluate a child's behavioral reaction to screen devices in early childhood.

## Introduction

Concerns regarding the impact of digital media use on the well-being of users, especially children, are long-standing. Most recently, with the increasing integration of digital screen devices into our everyday life, there has been increased attention to the risk of “problematic” use or pathological use, particularly as it relates to smartphones, video games, and internet use (1–3). Much of this is driven by concerns for the well-being of children and adolescents, who may be particularly susceptible to potential harms, given their ongoing development and the proven attention-grabbing technology used in such media (4–6). Problematic media use has been referred to as “excessive use that interferes with the child's functioning.” (7) While there is ongoing debate as to when problematic use is considered to be an addiction, growing evidence has elucidated negative consequences associated with problematic use, including impaired mental and physical health (2, 7–11). Some studies suggest up to 10% of children and adolescents have problematic screen use (11).

Because children start using screen devices in the first few years of life (12, 13), early identification of those at risk for future problematic use could inform prevention efforts in very early childhood (11). The Interactional Theory of Childhood Problematic Media Use developed by Domoff et al (7). draws from Bronfenbrenner's social ecological model (14), outlining numerous factors across multiple levels that may contribute to the development of problematic screen use, including distal (e.g., poverty) and proximal (e.g., parental media use, parenting practices, and beliefs) levels. Proximal child-level factors include child behavioral or emotional problems. Adding to this, findings by Coyne et al. suggest that child temperament (i.e., negative affect and effortful control) at ages 2 and 3 is associated with problematic screen use (15). Experts recognize, that at the individual child level, across both neurotypical and neurodivergent children (e.g., individuals with autism or attention deficit disorder), a differential susceptibility to media may exist, predicting media use and possibly its impact on child outcomes (7, 16–18). As outlined by Valkenburg et al. in the Differential Susceptibility to Media Effects Model (17), individuals may have different susceptibility to media effects, influenced by dispositional, developmental, and social factors. For example, individual differences in sensory processing, which may be biologically driven (19), may explain differences in children's susceptibility to the attention-grabbing techniques used in current-day digital content or the reinforcing effect of screen use implemented by parents to regulate a toddler's emotions (7, 20). Since problematic screen use takes time to develop, early identification of those susceptible to problematic use would offer the opportunity to intervene before problems develop.

Parents across varying child ages acknowledge that some children show a “strong desire” to use screen devices or are “attached” to their devices (21, 22). Parents may see such characteristics before any functional limitations occur, i.e., before the development of problematic screen use. Since screen use habits develop in early childhood (23), identifying children in the first few years of life who show a particular affinity or attraction to screen devices may be possible. In addition to possibly offering a way to identify children at higher risk for problematic use, a child's early affinity to screen devices may add to our current understanding of child screen use, going beyond those factors that are typically measured, such as duration of use, context of use, and content viewed.

To date, there has been no measure to identify children in the first few years of life who show a particular affinity to, or elevated interest in, screen devices. The Problematic Media Use Measure - Short Form (PMUM-SF) by Domoff et al. is one of the most widely used measures of problematic media use in children (11, 24). However, because it was developed for children 4–11 years old, this measure may not be relevant to the developmental stage of toddlers. Since toddlers do not have strong self-regulation and their language skills are developing, parents can, for the most part, completely control their toddler's screen use at home. Thus, a young child's media use may not yet “interfere with family activities” (except in extreme cases) and parents may not be able to report if screen media is all their child “thinks about,” as captured in the PMUM-SF. Aiming to capture a child's sensory behaviors as they relate to media, Harrison et al. developed the Child Media Sensory Curation Inventory, for children ages 3–14 years old. Again, items in this measure may not be as relevant for one and two year olds, such as whether the child “prefers 3D movies to regular movies”, gets a headache from watching 3D movies, or if the child turns down the sound when playing video games (18). A measure specific to toddlers that captures their affinity for screen devices is needed.

To address the gap in measurement, we developed survey measures of toddler affinity to screen media, defined as a child's attraction to screen devices. In our earlier work, parents reported some differences in beliefs and parenting practices by screen device type (25). Recognizing that a child's affinity may differ across device types, we aimed to develop separate measures of affinity to television and mobile devices. While the topic is applicable across all toddlers, we focused this work on Mexican American families with toddlers because little is known about the development of screen use behaviors in early childhood in Latino populations. Latino children, especially those living in low-income families, are exposed to more screen media compared to their non-Latino white peers (26–28). Moreover, screen-related outcomes, such as poor sleep and obesity, are more common in Latino children than non-Latino white children (29–31). Recognizing that subgroups exist within the Latino population, we focused on Mexican American families. Nearly two-thirds of Latinos in the US identify as having Mexican heritage (32).

## Materials and methods

### Study design

This study utilized an exploratory sequential mixed methods (qualitative -> quantitative) approach to develop measures of toddler affinity to screen devices. Instrument development was conducted as part of a larger study evaluating screen use in toddlers in Mexican American families. The study, which was conducted in the greater Denver metropolitan area, occurred in 2 phases, with findings from Phase 1 informing item development for Phase 2. Both Phases 1 and 2 were approved by the Colorado Multiple Institutional Review Board.

### Phase 1—item development

We conducted 32 semi-structured interviews with parents of toddlers 15–26 months old. Participants were Mexican American mothers (*n* = 22) and their partners (*n* = 10). The purpose of the interviews was to explore parental day-to-day management of toddler screen use. Methods for this work are described in detail elsewhere (25, 33). Briefly, from March 2019-April 2020, participants were recruited in the waiting room of a general pediatrics clinic located in a federally qualified health center that primarily serves families with incomes at or below the federal poverty level. Following informed consent, interviews were conducted in the preferred language of the participant by trained bilingual, bicultural Latina research assistants (RAs). Interviews were audio recorded and professionally transcribed.

#### Thematic analysis

Leveraging the richness of the parent interview data, we conducted a thematic analysis to identify patterns or themes about child affinity for screens. We chose this approach to accommodate both inductive and deductive analysis, acknowledge reflexivity and the interpretive processes of qualitative analysis, and allow a nuanced understanding of the participant perspective (34). To start, we familiarized ourselves with the interview transcripts, made notes about our insights, re-listened to the audio, and talked together about the interviews. From those notes, we began to generate inductive codes, supplementing the code list with codes based on our prior work (35, 36). Two RAs then independently coded each transcript, followed by discussions among the coders and a qualitative methodologist aimed at interpretation of meaning and consensus on code application. The inductive and deductive codes and their definitions were iteratively refined by the team. Through thematic analysis, coded text segments clustered together, forming a clear concept of affinity in parental perceptions of a child's attraction to screen devices. Attributes or characteristics of toddler affinity to screen devices included parents' descriptions of child behavior conceptually reflecting high affinity, such as “too attached”, “throws a tantrum” when cannot use a device, wants to use a smartphone as soon as child sees it, as well as comments reflecting low affinity, such as child “doesn't pay much attention” to a device, prefers parental attention or to “be playing with his toys” rather than using a screen device.

#### Items

We developed 20 items reflecting parental expressions of child affinity to screen devices, using parents' wording as much as possible. Items were designed to reflect high or low affinity. The four-point response options were disagree (0), sort of disagree (1), sort of agree (2), and agree (3). We limited the response scale to four options based on evidence suggesting that more than four options can be challenging for individuals with low literacy (37).

#### Translation

A bilingual/bicultural team member translated items into Spanish or English as needed. The two language versions were reviewed side by side by a group of bilingual investigators and research staff, evaluating for conceptual equivalence and contextual and cultural relevance. We applied a decentering process in which alterations were made to either language version to obtain conceptual equivalence (38, 39).

#### Field pretesting

Items were pre-tested in cognitive interviews in both Spanish and English to ensure easy comprehension as well as shared conceptual meaning across participants and the investigative team (40). Interviewers read each item out loud and asked participants to respond. Participants were then asked to restate the item in their own words, followed by probes to evaluate meaning, and ease of responding. Participant responses for each item were evaluated by a committee of investigators and research staff using an iterative process. Adjustments were made to problematic wording, and items were retested until no additional issues were identified. Through this process, some items were dropped and others were revised. Among remaining items, we identified those that performed best in pretesting and reflected either high or low affinity. During cognitive interviews, participant comments reinforced the concept that affinity may vary by device type; thus, items were then altered to replace the term “screen devices” with either “TV” or “mobile devices” (inclusive of smartphone and tablet use), resulting in 10 items applicable to both device groups, and 2 additional items specific to mobile devices for a total of 22 items.

### Phase 2—quantitative scale development

#### Sample

The 22 items developed in Phase 1 were administered as part of the larger study enrolling Mexican American mothers, their partners, and their toddlers ages 15–26 months old, recruited from a large safety net health system in the greater Denver metropolitan area in Colorado.

#### Study procedures

Data collection occurred over a 3-year period from November 2020 to December 2023. Following informed consent, mothers were administered two 1–1.5 h phone surveys by trained bilingual bicultural RAs. Given the prevalence of literacy challenges in Latino communities, we orally administered survey items to all participants (41, 42). Surveys occurred approximately 7–10 days apart. In between phone visits, parents completed 7-day activity diaries. Data were collected and managed using REDCap electronic capture tools (43).

#### Measures

Two affinity measures were administered: Affinity-TV (10 items) and Affinity-Mobile (12 items). Affinity-TV items were only administered to parents who reported their child had used a TV, and similarly Affinity-Mobile items were only administered to parents who reported their child had ever used a mobile device. Due to an issue in REDCap, one of the Affinity-TV items was not administered to 25 participants. These data can be considered missing completely at random (44). All available data were used in analyses. Demographics were collected via survey items. Acculturation was measured using the Bidimensional Acculturation Scale for Hispanics consisting of two scales: Hispanic acculturation (12 items) and non-Hispanic acculturation (12 items). This measure has been validated in Mexican Americans (*α* = .93; *α* = .97 respectively) (45). Scores range from 1 to 4, with higher scores on each scale reflecting higher acculturation to the named cultural domain. The Brief Infant–Toddler Social and Emotional Assessment was also administered as a measure of social-emotional/behavioral problems (31 items, *α* = 0.79) and competencies (11 items, *α* = 0.65) (46). Using age-adjusted cut-scores in the Examiner's manual (47), we created two variables: high problem behaviors and low social-emotional competencies. Across all survey items, participants could abstain from answering or answer “don't know” if relevant. Mothers completed a 7-day pen and paper screen use diary, which was used to calculate daily average minutes of toddler television use and mobile device use. Duration of television and mobile devices (inclusive of smartphone and tablet use) were calculated for those with 5 or more completed diary days. Diary measurement of screen use is highly correlated with actual child screen use (48, 49). Moreover, a study by Mendoza et al. support the reliability and feasibility of using diaries in low-income Latino families (50).

#### Analysis

The full sample of mothers (*n* = 384) who completed the study was randomly divided into two subsamples for measurement evaluation, one for Exploratory Factor Analysis (EFA) (*n* = 192) and one for Confirmatory Factor Analysis (CFA) (*n* = 192), stratified based on partner participation. Separate EFA models were estimated for Affinity-TV items and for Affinity-Mobile items, using maximum likelihood estimation and promax (oblique) rotation. Items with wording in the opposite direction as others were reverse coded prior to modeling. Models with 1–4 factors were evaluated. The final model was selected based on the observed scree plot of eigenvalues, model interpretability, and model convergence. Once the final factor structure was selected, items with factor loadings ≤0.35 were removed. The Cronbach's alpha of each factor was calculated as a measure of internal consistency reliability. CFA models were estimated using the Affinity-TV items and Affinity-Mobile items retained from EFA. CFA model fit was assessed using the comparative fit index (CFI ≥ 0.9) and root mean square error of approximation (RMSEA ≤ 0.08). In the event of CFA misfit, modification indices were considered. Analyses were completed using SAS Version 9.4 (EFA and correlations) and MPlus Version 8.6 (CFA).

## Results

### Sample characteristics

Mothers in Phase 2 were, on average, 31.4 (SD = 6.0) years old, with 11.6 (SD = 2.5) years of education. Most women (85%) had a partner and preferred to be interviewed in Spanish (77%). Children were, on average, 21.2 (SD = 3.1) months old and about half were male (49%). Maternal and child characteristics are included in Table 1, along with behavioral factors. No participant answered “Don't Know” or abstained from answering the Affinity-TV or Affinity-Mobile items, resulting in complete data for TV users and mobile device users, aside from the aforementioned REDCap issue.

**Table 1:** Characteristics of a sample of Mexican American mothers with toddlers ages 15-26 months (*n* = 384).

Characteristic	Whole sample (*n* = 384) Mean (SD), median (IQR),^a^ or % (n)	EFA (*n* = 192) Mean (SD), median (IQR),^a^ or % (n)	CFA (*n* = 192) Mean (SD), median (IQR),^a^ or % (n)
Mother			
Education (years)	11.6 (2.5)	11.4 (2.6)	11.8 (2.4)
Partnered (%)	85% (*n* = 328)	88% (*n* = 168)	83% (*n* = 160)
Age (years)	31.4 (6.0)	31.1 (6.1)	31.8 (5.8)
Acculturation: Hispanic	3.6 (3.3–3.9)	3.5 (3.3–3.9)	3.7 (3.3–3.9)
Acculturation: non-Hispanic	2.3 (1.7–3.2)	2.2 (1.6–3.3)	2.3 (1.7–3.1)
Language of interview: Spanish	77% (*n* = 296)	77% (*n* = 148)	77% (*n* = 148)
Child			
Age (months)	21.2 (3.1)	21.2 (3.1)	21.1 (3.2)
Male Sex (%)	49% (*n* = 189)	52% (*n* = 100)	46% (*n* = 89)
Child Behavioral Factors			
Daily TV use (minutes)	92.1 (49.3–169.3)	90.0 (50.7–174.6)	98.6 (47.1–167.1)
Daily mobile device use (minutes)	21.4 (4.3–57.7)	24.3 (4.3–54.6)	19.3 (4.3–62.1)

^a^ Mean and standard deviation (SD) presented for approximately normally distributed variables; Median and interquartile range (IQR) presented for skewed variables.

EFA, exploratory factor analysis; CFA, confirmatory factor analysis.

### Exploratory factor analysis

Factor loadings and Cronbach's alphas for Affinity-TV and Affinity-Mobile are presented in Table 2. The one-factor solution for Affinity-TV was selected (Eigenvalue = 4.23). No items were removed due to low factor loadings, with loadings ranging from 0.44 to 0.66. The one-factor solution for Affinity-Mobile was also selected (Eigenvalue = 9.55), with factor loadings ranging from 0.51 to 0.74.

**Table 2:** Exploratory factor analysis (EFA) loadings for Affinity-TV and Affinity-Mobile^c^ (*n* = 192).

Items	EFA factor loadings
Affinity-TV	
TV1. (CHILD) loses interest in the TV quickly.^a^	0.45
TV2. (CHILD) throws a temper tantrum if you turn off the TV.	0.51
TV3. (CHILD) prefers to be playing with toys instead of watching TV.^a^	0.51
TV4. Programs on the TV really get (CHILD)'s attention.	0.48
TV5. The TV holds (CHILD)'s attention for only a very short time.^a^	0.55
TV6. (CHILD) is very attached to the TV.	0.66
TV7. (CHILD) would prefer to watch TV rather than play with other people.	0.46
TV8. (CHILD) would rather be with you than watch TV. ^a^	0.44
TV9. (CHILD) stays calm if you tell her/him s/he cannot watch TV. ^a^	0.51
TV10. (CHILD) really enjoys watching TV.	0.57
Affinity-TV scale mean (SD)^b^	0.79 (0.51)
Cronbach's alpha	0.78
Affinity-Mobile	
M1. (CHILD) loses interest in mobile devices quickly.^a^	0.61
M2. (CHILD) throws a temper tantrum if you take away a mobile device	0.68
M3. (CHILD) prefers to be playing with toys instead of using mobile devices.^a^	0.72
M4. Programs on mobile devices really get (CHILD)'s attention.	0.51
M5. Mobile devices hold (CHILD)'s attention for only a very short time.^a^	0.67
M6. (CHILD) is very attached to mobile devices.	0.69
M7. If (CHILD) sees the phone, s/he won't do anything else until s/he gets to use it.	0.60
M8. (CHILD) would prefer to use mobile devices rather than play with other people.	0.66
M9. (CHILD) would rather be with you than use a mobile device.^a^	0.61
M10. (CHILD) stays calm if you tell her/him s/he cannot use a mobile device.^a^	0.74
M11. (CHILD) wants to use your phone the moment s/he sees it.	0.58
M12. (CHILD) really enjoys using mobile devices.	0.64
Affinity-Mobile scale mean (SD)	0.99 (0.68)
Cronbach's alpha	0.89

Response options were coded 0–3.

^a^ Reverse coded.

^b^ SD, standard deviation.

^c^ Scales can be used for research purposes if this paper is cited, and corresponding author is notified prior to use.

### Confirmatory factor analysis

The one-factor CFA model for Affinity-TV indicated acceptable fit (CFI = 0.92, RMSEA = 0.068), with a Cronbach's alpha of 0.80 and a mean score of 0.8 (SD = 0.53). In the Affinity-Mobile CFA, model fit did not meet acceptable criteria (CFI = 0.81, RMSEA = 0.11). Modification indices were inspected and after allowing correlations between select item residuals (Items 1 and 5; 2 and 5; 2 and 10; and, 7 and 8), model fit was acceptable (CFA = 0.90, RMSEA = 0.083). Cronbach's alpha for Affinity-Mobile was 0.85, with a mean score of 0.8 (SD = 0.60).

### Scale scores and correlations

Scale scores were calculated as the mean of the included items. Spearman correlations were estimated between the Affinity scales and demographic and behavioral characteristics using the full, combined sample (Table 3). There was a significant positive correlation between Affinity-TV and Affinity-Mobile (rs = 0.44, *p* < 0.001). Affinity-TV was significantly positively correlated with toddler average daily minutes of TV use (rs = 0.27, *p* < 0.001) and with toddler average daily minutes of mobile use (rs = 0.10, *p* < 0.05). Affinity-Mobile was significantly positively correlated with toddler average daily minutes of mobile use (rs = 0.31, *p* < 0.001), but not with toddler average daily minutes of TV (rs = −0.04, NS). Greater Affinity-TV was correlated with low social-emotional competencies (rs = 0.15, *p* < 0.05). Greater Affinity-Mobile was correlated with both high levels of behavioral problems (rs = 0.22, *p* < 0.01) and low social-emotional competencies (rs = 0.23, *p* < 0.001). The only significant correlation involving maternal demographics was between Affinity-TV and maternal age (rs = −0.13, *p* < 0.05), with older mothers reporting that their toddler had lower Affinity-TV scores. Finally, greater Affinity-TV and Affinity-Mobile were both correlated with older child age (rs = 0.15, *p* < 0.01 and rs = 0.22, *p* < 0.001 respectively), but not with child sex.

**Table 3:** Spearman correlations between Affinity-TV, Affinity-Mobile and demographic and behavioral variables (*n* = 305–384).

Variables	Affinity-TV (*n* = 384)	Affinity-Mobile (*n* = 305)
Demographic variables		
Mother		
Education (years)	0.02	0.01
Partnered	−0.05	−0.09
Age (years)	−0.13*	0.01
Acculturation: Hispanic	−0.03	−0.03
Acculturation: Non-Hispanic	0.07	−0.02
Interview language: Spanish	−0.07	0.06
Child		
Age (months)	0.15*	0.22**
Sex (male)	−0.02	0.04
Child Behavioral Variables		
Average daily TV use (minutes)	0.27***	−0.04
Average daily mobile device use (minutes)	0.10*	0.31***
Low social-emotional competencies	0.15*	0.23***
High social-emotional/behavioral problems	0.13	0.22**

* *P* < .05.

** *p* < .01.

*** *p* < .001.

## Discussion

The purpose of this study was twofold: to develop measures of toddler affinity to screen devices (Affinity-TV and Affinity-Mobile), and evaluate their reliability and validity in a sample of Mexican American families with toddlers. We report strong internal consistency. Findings also support adequate predictive validity for both measures. These findings support the use of Affinity-TV and Affinity-Mobile in toddlers as measures of a child's attraction to screen devices. Reports suggest that about 40% of parents of children 0–8 years old worry about their child's potential for addiction to screen devices, specifically mobile devices (51). These measures may help to identify early risk for problematic use. Moreover, as experts have called for research that goes beyond evaluations of screen use duration (16), these measures offer a novel way to evaluate a child's behavioral reaction to screen devices in early childhood.

We propose that a child's affinity to screen devices is a conceptually cohesive and measurable characteristic of a toddler's interaction with screen devices, with higher scores possibly indicating a susceptibility to problematic screen use. While this susceptibility hypothesis has not yet been tested, evidence suggests that children may have differential susceptibility to problems with screen devices, influenced by both genetic and environmental factors (52–54). Neuroscientific evidence in humans and animals, including neurochemical, genetic, and neuroanatomical findings, support the concept of a predisposition to problematic use (52, 53, 55, 56). Harrison et al. propose that individual-level differences in underlying sensory preferences within both neurotypical and neurodivergent individuals, may explain the differential susceptibility of children to digital media (18). In a sample of 3–14 year olds, they found that a child's sensory media preferences (e.g., loud volume, fast movements) were associated with problematic screen use (18, 54). Environmental factors (e.g., inadequate caregiving, traumatic experiences) are known to play an important role in the development of addictive behaviors, in part due to the interplay between genes and the environment (57–59). Both genetic and environmental factors may therefore contribute to a child's susceptibility to problematic screen use. Consistent with this research, we found that low social emotional competencies and high levels of behavioral problems were correlated with higher levels of affinity to screen use, supporting the validity of the two measures. Observational methods of toddler interactions with screen devices could further validate parental report of child affinity. Moreover, longitudinal studies are needed to evaluate the relationship between child affinity to screen devices and the development of problematic screen use in later years. Research identifying the precursors to child affinity, including child-level factors and environmental contributors, could also facilitate the development of clinical interventions.

Underscoring the important role of the environment on screen use in children, multiple existing frameworks emphasize the role that context plays in the development of problematic screen use (7, 16–18). Domoff's Interactional theory of childhood problematic media use highlights parent and family level factors as well as the digital environment design (e.g., design features prolonging engagement) as possibly contributing to problematic media use (7). Barr et al's Dynamic, Relational, Ecological Approach to Media Effects Research (DREAMER) framework stresses the influence of family and structural factors in influencing child screen use, including problematic use (16). Extending these frameworks to the concept of affinity to screen devices, we suggest that contextual factors may contribute to a child's affinity, starting simply with exposure to media at particular points in development. Children's exposure to media, and the characteristics of the media environment around them, probably contribute to their affinity to TV and/or mobile devices. Our findings that Affinity-TV and Affinity-Mobile are correlated with child screen use duration support this notion. Parenting practices related to screen use, such as whether screen devices are used to calm a young child, reinforcing a child's sense of “need” for such devices, probably also play a role in developing an affinity to screen use. Clearly, identification of environmental contributors to child affinity is an important area for evaluation.

An important quality of these affinity scales is that they are device-specific measures, one for TV and one for mobile devices, allowing for identification of differences in the way children relate to TV vs. mobile devices. Many measures of screen use are limited in that they either capture screen use broadly or focus on one device type (60). While TV is widely used by toddlers, exposure to mobile devices has increased rapidly over the last decade, with studies suggesting that most children start using mobile devices in the first year of life (61–63). Yet, for some children, mobile device use is limited in the first few years of life (64). The need for two measures was driven by our formative work suggesting that parents often perceive TV and mobile device use differently in this age group (25). Affinity for each type of screen may therefore differ. Additional research is needed to understand factors contributing to affinity across device types, including use of different devices and device-specific parenting practices.

These measures of affinity to screen use have numerous strengths. To start, the focus on children of Mexican heritage, who represent about 15% of children in the US (65, 66), ensures that the measures are relevant for this population. The grounding of these measures in findings from semi-structured interviews and the further refinement of items with cognitive interviews helps to ensure their relevance and validity for this population. Finally, we developed two language versions, using best practice methods for translation to allow for use in both languages. Numerous health outcomes disparately affect the Latino community, warranting the need for research focused on this population. For example, childhood obesity affects 16% of Latino preschoolers compared to 5% of their non-Latino white peers (31). Since screen use is associated with childhood obesity, focused research on screen use in this population is needed in order to inform the development of culturally- and contextually-relevant interventions.

Limitations of this work warrant mention. Since this work was conducted in a specific population in one metropolitan area with participants recruited from a clinic system serving mainly low-income families, the generalizability to other populations, socioeconomic strata, and settings will need to be tested. Additionally, because the correlational findings are based on cross-sectional data, the directionality of the tested relationships is unknown. Further evaluation is needed, including examining whether the relationships between screen use and affinity are bidirectional. Because most of the sample was interviewed in Spanish, we were unable to test for measurement invariance or validity across languages, which should be evaluated in future work. While our use of a four-point response scale was intentional, it may have limited the scale's sensitivity for measuring the entire range of affinity levels. Future research could explore this, while balancing ease of response for individuals with low literacy. Finally, it is possible that the oral administration of survey items influenced participant responses. Use of audio computer-assisted self-interviews (ACASI) could be considered in future research as an alternative method to overcome literacy issues while also minimizing possible social desirability bias (67).

## Conclusions

In summary, we present two novel scales, Affinity-TV and Affinity-Mobile, that can be used to measure a toddler's attraction to screen devices. These measures capture an aspect of screen use that goes beyond the duration of use, and that might facilitate early identification of those toddlers at risk for the development of problematic screen use. Future studies in other populations are needed, as well as longitudinal evaluations investigating individual-level and contextual contributors to child affinity. Ultimately, such investigations could lead to the development of interventions that could promote healthy screen use in toddlers.

## Data Availability

The datasets presented in this article are not readily available because a data sharing agreement is required. Requests to access the datasets should be directed to Darcy Thompson, darcy.thompson@cuanschutz.edu.
